# The Antidiabetic Effects and Modes of Action of the *Balanites aegyptiaca* Fruit and Seed Aqueous Extracts in NA/STZ-Induced Diabetic Rats

**DOI:** 10.3390/pharmaceutics14020263

**Published:** 2022-01-22

**Authors:** Asmaa S. Zaky, Mohamed Kandeil, Mohamed Abdel-Gabbar, Eman M. Fahmy, Mazen M. Almehmadi, Tarek M. Ali, Osama M. Ahmed

**Affiliations:** 1 Biochemistry Department, Faculty of Science, Beni-Suef University, Beni-Suef P.O. Box 62521, Egypt; asmaa_zaky221@yahoo.com (A.S.Z.); hhmgabar@yahoo.com (M.A.-G.); 2 Biochemistry Department, Faculty of Veterinary Medicine, Beni-Suef University, Beni-Suef P.O. Box 62521, Egypt; mohamed.kandeal@vet.bsu.edu.eg; 3 Department of Internal Medicine, Faculty of Medicine, Helwan University, Helwan 11795, Egypt; dremf@hotmail.com; 4 Department of Clinical Laboratory Sciences, College of Applied Medical Sciences, Taif University, P.O. Box 11099, Taif 21944, Saudi Arabia; dr.mazen.ma@gmail.com; 5 Department of Physiology, College of Medicine, Taif University, P.O. Box 11099, Taif 21944, Saudi Arabia; tarek70ali@gmail.com; 6 Physiology Division, Department of Zoology, Faculty of Science, Beni-Suef University, Beni-Suef P.O. Box 62521, Egypt

**Keywords:** NA/STZ-induced diabetes mellitus, *Balanitis aegyptiaca*, fruit, seed, aqueous extracts

## Abstract

Diabetes mellitus (DM) is a chronic metabolic disorder that threatens human health. Medicinal plants have been a source of wide varieties of pharmacologically active constituents and used extensively as crude extracts or as pure compounds for treating various disease conditions. Thus, the aim of this study is to assess the anti-hyperglycemic and anti-hyperlipidemic effects and the modes of action of the aqueous extracts of the fruits and seeds of *Balanites aegyptiaca* (*B*. *aegyptiaca*) in nicotinamide (NA)/streptozotocin (STZ)-induced diabetic rats. Gas chromatography–mass spectrometry analysis indicated that 3,4,6-tri-O-methyl-d-glucose and 9,12-octadecadienoic acid (Z,Z)- were the major components of the *B*. *aegyptiaca* fruit and seed extracts, respectively. A single intraperitoneal injection of STZ (60 mg/kg body weight (b.w.)) 15 min after intraperitoneal NA injection (60 mg/kg b.w.) was administered to induce type 2 DM. After induction was established, the diabetic rats were treated with the *B*. *aegyptiaca* fruit and seed aqueous extracts (200 mg/kg b.w./day) via oral gavage for 4 weeks. As a result of the treatments with the *B*. *aegyptiaca* fruit and seed extracts, the treated diabetic-treated rats exhibited a significant improvement in the deleterious effects on oral glucose tolerance; serum insulin, and C-peptide levels; liver glycogen content; liver glucose-6-phosphatase and glycogen phosphorylase activities; serum lipid profile; serum free fatty acid level; liver lipid peroxidation; glutathione content and anti-oxidant enzyme (glutathione peroxidase, glutathione-S-transferase, and superoxide dismutase) activities; and the mRNA expression of the adipose tissue expression of the insulin receptor β-subunit. Moreover, the treatment with fruit and seed extracts also produced a remarkable improvement of the pancreatic islet architecture and integrity and increased the islet size and islet cell number. In conclusion, the *B*. *aegyptiaca* fruit and seed aqueous extracts exhibit potential anti-hyperglycemic and anti-hyperlipidemic effects, which may be mediated by increasing the serum insulin levels, decreasing insulin resistance, and enhancing the anti-oxidant defense system in diabetic rats.

## 1. Introduction

Diabetes mellitus (DM) is a chronic metabolic syndrome with a number of different etiologies. It severely affects the life of patients and heightens the risk of developing other diseases [1]. It is characterized by abnormal carbohydrate, lipid, and protein catabolism and anabolism due to insulin resistance or hypoinsulinism [2]. The recent statistics from the International Diabetes Federation (IDF) indicated that approximately 463 million adults between the ages of 20 and 79 years have diabetes, most of whom live in poor and developing countries, and this is expected to increase to 700 million by 2045 [3]. Many factors contribute to this increasing prevalence of DM, including population growth, urbanization, nutritional transition, physical inactivity, and dietary change [4,5].

Although the existing antidiabetic synthetic drugs have several benefits, they are accompanied by many adverse side effects [6]. Thus, alternative antidiabetic agents with less or no hazardous side effects are needed [6,7,8]. Recently, new active medicines have been extracted from plants and have antidiabetic activity with more effectiveness than oral chemical hypoglycemic drugs used in proven therapy [9]. Medicinal plants contain various bioactive compounds that have multiple activities in insulin production, insulin action, or both [10]. 

Eskander and WonJun described several types of Egyptian plant and herb prescriptions for the treatment of DM, and these belong to various families [11]. *Balanites aegyptiaca* (L.) Delile, which belongs to the Zygophyllaceae family, is used traditionally in African countries as an antihelmintic and in the treatment of jaundice [12,13]. In Egyptian folkloric medicine, the fruit is used as an oral anti-hyperglycemic agent [14], and herbalists in the Egyptian market sell the fruits as an antidiabetic agent. Nevertheless, the quality control of such herbal products remains a great challenge. The aqueous extract of the mesocarp of the *B*. *aegyptiaca* fruit exhibited antidiabetic activities in streptozotocin (STZ)-induced diabetic mice and rats [14,15], and several saponins were isolated from the mesocarp [14,16,17]. Moreover, the *B*. *aegyptiaca* seed kernel contains a high amount of oil and protein, which differs from one source to another [18].

Therefore, the aims of this study are to evaluate the effects of aqueous extracts of the *B. aegyptiaca* fruit and seed on the glycemic state and lipid profile and to indicate their probable modes of action in nicotinamide (NA)/STZ-induced diabetic rats.

## 2. Materials and Methods

### 2.1. Chemicals

NA, STZ [2-deoxy-2-(3-methyl-3nitrosoureido)-D-glycopyranoside], glucose-6-phosphate, glucose-1-phosphate, anthrone, reduced glutathione (GSH), malondialdehyde (MDA), and 1-Chloro-2,4-dinitrobenzene were purchased from Sigma-Aldrich Chemical Co., St Louis, MO, USA. All other chemicals were of analytical grade and were obtained from standard commercial supplies.

### 2.2. Experimental Animals

Male Wistar rats weighing approximately 110–140 g were used as experimental animals in the present study. The rats were housed in standard polypropylene cages and placed under a regulated room temperature of 22 ± 2 °C and humidity of 55 ± 5% with a 12:12 light–dark cycle. They were fed with a standard diet of known composition and water *ad libitum*. All animal procedures were in accordance with the ethical guidelines for the use and care of animals of the Experimental Animal Ethics Committee, Faculty of Science, Beni-Suef University, Egypt (Ethical Approval Number: BSU/FS/2015/17). All attempts were made to minimize the number and pain of used animals.

### 2.3. Induction of DM

After fasting for 16 h, DM was experimentally induced in male Wistar rats via an intraperitoneal (IP) injection of 60 mg NA/kg body weight (b.w.) to 16-h fasted rats before the IP injection of 60 STZ mg/kg b.w. [19]. The rats were tested for serum glucose levels 10 days after STZ was injected. The overnight-fasted (10–12 h) animals were given glucose (3 g/kg b.w) via an intragastric tube. The blood samples were taken from the lateral tail vein after 2 h of oral administration, left to coagulate, and centrifuged. The serum glucose level was then measured. The experiment included rats with a serum glucose level between 180 and 300 mg/dL, after 2 h of glucose intake, whereas the others were excluded.

### 2.4. Preparation of the B. aegyptiaca Fruit and Seed Aqueous Extracts

The *B*. *aegyptiaca* fruits and seeds were powdered using an electrical grinder. The fruit or seed powders were infused in boiled distilled water (200 mg/10 mL) for 15 min. The obtained extracts were filtered pending their use via oral gavage.

### 2.5. Gas Chromatography–Mass Spectrometry (GC-MS) Analysis

Both the *B*. *aegyptiaca* fruit and seed aqueous extracts were phytochemically analyzed via GC-MS (Producer, City, Country) according to the method described in our previous publication [20].

### 2.6. Experimental Design and Blood and Tissue Sampling

**Scheme 1:**
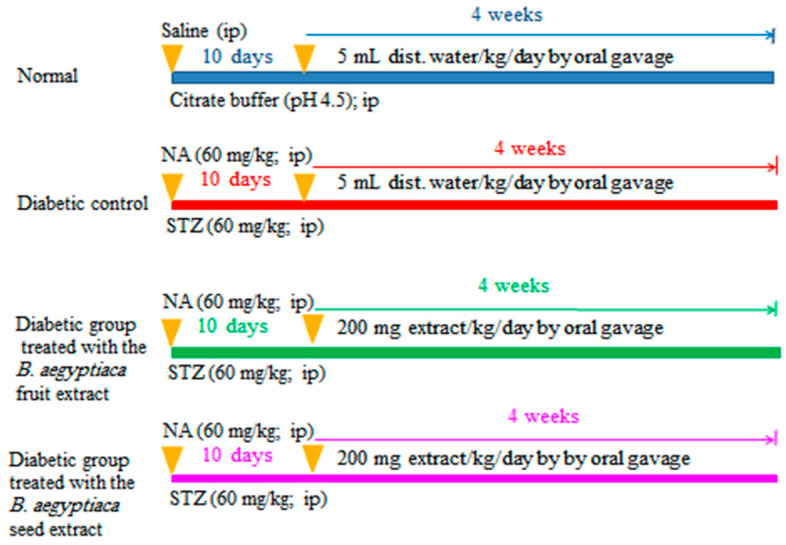
Schematic figure showing the experimental design and animal grouping. NA: nicotinamide; STZ: streptozotocin; *B. aegptiaca*: *Balanites aegptiaca*; and ip: intraperitoneal.

The rats were allocated into four groups of six rats (Scheme 1):

Group I (Normal group): This group was assigned as the normal control group, and rats included in this group was given distilled water daily (5 mL/kg b.w./day) via oral gavage for 4 weeks.

Group II (Diabetic control): This group was assigned as the diabetic control group, and the diabetic rats within this group were given distilled water daily (5 mL/kg b.w./day) via oral gavage for 4 weeks.

Group III (Diabetic rats treated with the *B*. *aegyptiaca* fruit extract): This group consisted of diabetic rats that were treated daily with *B*. *aegyptiaca* fruit extract at a dose level of 200 mg/kg b.w./day via oral gavage for 4 weeks.

Group IV (diabetic rats treated with the *B*. *aegyptiaca* seed extract): This group consisted of diabetic rats that were treated daily with *B*. *aegyptiaca* seed extract at a dose level of 200 mg/kg b.w./day via oral gavage for 4 weeks.

At the day before sacrifice, oral glucose tolerance (OGT) test (OGTT) was performed by administering glucose solution (3 g/kg b.w.) to overnight-fasted rats via oral gavage. Successive blood samples were then obtained at 0, 30, 60, 90, and 120 min. Blood samples were left to coagulate and centrifuged. Sera were separated via centrifugation at 3000 rpm for 15 min, and the serum glucose levels were determined. One day after the end of the experiment, blood samples were collected from the jugular vein under diethyl ether inhalation. Moreover, the rats were euthanized and dissected for the excision of various organs.

### 2.7. Blood Sampling and Tissue Sampling

The blood obtained from the jugular vein of each rat was left to coagulate at room temperature. Serum was separated for 15 min via centrifugation at 3000 rpm and stored at −20 °C pending its use for the determination of insulin and C-peptide levels as well as other biochemical parameters. The rats were rapidly dissected. Visceral adipose tissues were excised and kept at −70 °C until use for ribonucleic acid (RNA) extraction and the detection of the messenger RNA (mRNA) of adiponectin and resistin via reverse transcription-polymerase chain reaction (RT-PCR). The liver was excised for the determination of oxidative stress parameters and glycogen content and glycogen metabolizing enzymes. The pancreas was also excised for histological investigation.

### 2.8. Biochemical Analysis

The serum glucose levels were measured using the reagent kits purchased from Spinreact Company (Spain) using the method of Trinder et al. [21]. The serum insulin level was measured using sandwich enzyme-linked immunosorbent assay (ELISA) using kits purchased from Linco Research, USA, in accordance with the manufacturer’s instructions. Similarly, the serum C-peptide level was measured using the ELISA kits purchased from Linco Research, USA, in accordance with the manufacturer’s instructions. Homeostatic model assessment (HOMA)-insulin resistance (IR), HOMA-insulin sensitivity (IS) [22], and HOMA-β cell function [23] were calculated using the following formulas, respectively:

HOMA-IR = (fasting insulin [µIU/mL] × fasting glucose [mg/dL])/405

HOMA-IS = 10,000/(fasting insulin [µIU/mL] × fasting glucose [mg/dL])

HOMA-β cell function = (20 × fasting insulin [µIU/mL])/(fasting glucose [mg/dL/18] − 3.5).

The liver glycogen content was measured using the method of Seifter et al. [24]. The liver glucose-6-phosphatase and glycogen phosphorylase activities were measured using laboratory-prepared chemicals and the methods of Begum et al. [25] and Stallman and Hers, respectively [26]. The serum cholesterol level was assayed using the method of Allain et al. and the reagent kits purchased from Spinreact Company (Spain) [27]. 

The serum triglyceride level was determined using the reagent kit purchased from Reactivos Spinreact Company (Girona, Spain) and Fossati and Prencipe’s method [28]. The serum high density lipoprotein (HDL)-cholesterol level was measured using the method of Allain et al. (1974) and the reagent kit obtained from Spinreact Company, Spain [27]. The serum low density lipoprotein (LDL) cholesterol level was calculated using the formula of Friendewald et al. [29]:

LDL cholesterol = total cholesterol − triglycerides/5 − HDL cholesterol

Serum very low density lipoprotein (vLDL)-cholesterol was calculated using Norbert’s formula [30]:

vLDL cholesterol conc. = triglycerides/5

The serum free fatty acid (FFA) level was determined using Duncombe’s method [31].

### 2.9. RNA Isolation and RT-PCR

RNA was isolated from visceral adipose tissue using the GeneJet RNA purification kit produced by Thermo Fisher Scientific Inc., Branchburg, NJ 08876, USA, according to the procedures of Chomzynski and Sacchi [32] and Boom et al. [33]. The isolated RNA was quantified and qualified [34,35]. Thermo Scientific Verso 1-Step RT-PCR ReddyMix was used to produce cloned DNA that was amplified in the presence of specific forward and reverse primers using a Techne thermal cycler, Cole-Parmer, IL 60061, USA [35]. The primer pair sequences for the insulin receptor β-subunit were F: 5′CTGGAGAACTGCTCGGTCATT3′ and R: 5′GGCCA-TAGACACGGAAAAGAAG3′ [36], and those for β-actin re F: 5′TCACCCTGAAGTACCCCATGGAG3′ and R: 5′TTGGCCTTGGGGTTCAGGGGG3′ [37,38].

### 2.10. Determination of Oxidative Stress and Anti-Oxidant Defense Parameters

The glutathione content (GSH) in the liver was determined [39]. Moreover, glutathione-S-transferase (GST) activity in the liver was measured using Mannervik and Gutenberg’s method [40]. The liver glutathione peroxidase (GPx) activity was determined using the method of Matkovics et al. [41]. The superoxide dismutase (SOD) and lipid peroxidation (LPO) were determined using the methods of Marklund and Marklund [42] and Preuss et al. (1998), respectively [43].

### 2.11. Histological Investigation

The pancreas from each rat was rapidly excised after dissection and then fixed in 10% neutral buffered formalin for 24 h. The organs were routinely processed and sectioned at a thickness of 4 to 5 µm. The sections of the pancreas were stained with hematoxylin and eosin [44,45].

### 2.12. Statistical Analysis

The results were analyzed using the PC-STAT Program [46]. One-way analysis of variance (ANOVA) was followed by the least significant difference (LSD) test to compare various groups. Data were described as the mean ± SE. A *p* value of >0.05 was considered nonsignificantly different, whereas *p* values of <0.05 and <0.01 were considered significant and highly significant, respectively.

## 3. Results

### 3.1. GC-MS Analysis

**Figure 1:**
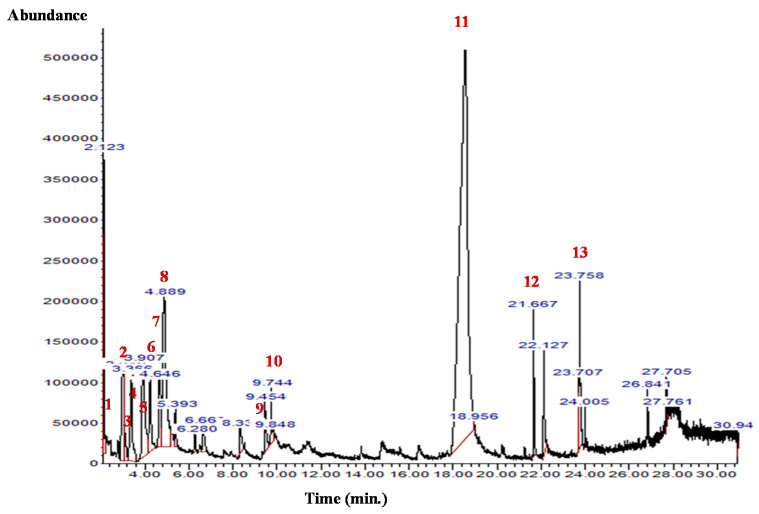
GC-MS chromatogram of the *B*. *aegyptiaca* fruit aqueous extract. 1: Hydrazine, 1,1-dimethyl-; 2 and 3: Butanal, 2-methyl-; 4: Propanoic acid, propyl ester; 5: Glyceraldehyde; 6: 2-Furanmethanol; 7: Isopropyl isothiocyanate; 8: Triethylphosphine; 9: 2,4(3H,5H)-Furandione; 10: Pyridine, 4-chloro-2,6-dimethyl-; 11: 3,4,6-Tri-O-methyl-d-glucose; 12: Perhydrohistrionicotoxin-2-thione, 2-depentyl-; and 13: Propyl 11,12-methylene-octadecanoate.

**Figure 2:**
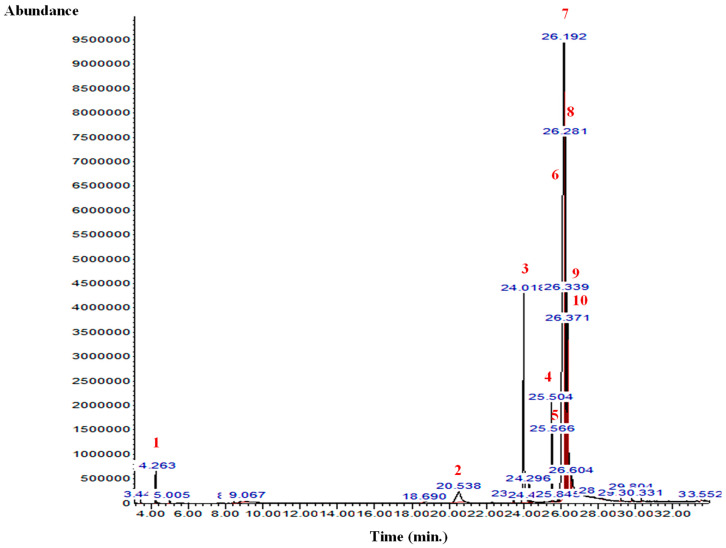
GC-MS chromatogram of the *B*. *aegyptiaca* seed aqueous extract. 1: Butane, 2-methyl-; 2: 3,4,6-Tri-O-methyl-d-glucose; 3: 3,4,6-Tri-O-methyl-d-glucose; 4: 9,12-Octadecadienoic acid (Z,Z)-; 5: 13-Octadecenoic acid, methyl ester; 6: 9,12-Octadecadienoic acid (Z,Z)-; 7: 8-Dodecen-1-ol, (Z)-; 8: 9,12-Octadecadienoic acid (Z,Z)-, 2,3-dihydroxypropyl ester; 9: Octadec-9-enoic acid; and 10: Benzoic acid, 4-(4-hydroxybenzylidenamino)-, propyl ester.

The GC-MS analysis of the *B*. *aegyptiaca* fruit and seed extracts showed the presence of several phytocomponents. Table 1 and Table 2 and Figure 1 and Figure 2 show the identified phytocomponents with their retention time, which was expressed as the peak area %. In the fruit extract, compounds 3,4,6-tri-O-methyl-d-glucose (52.55%) and triethylphosphine (9.31%) were the most abundant. Conversely, in the seed extract, compounds 9,12-octadecadienoic acid (Z,Z)- (38.27%), 8-dodecen-1-ol, (Z)- (15.09%), 2,3-dihydroxypropyl ester (11.47%), and H-cyclopenta [b]quinoxaline-1,2,3trione (11.39%) were the most abundant.

### 3.2. Effects on OGT

**Figure 3:**
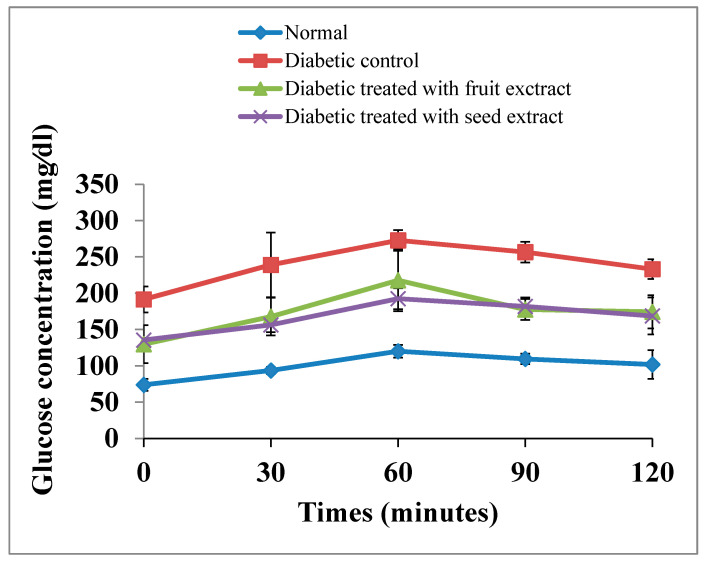
Effects of the *B*. *aegyptiaca* fruit and seed extracts on OGTT in NA/STZ-induced diabetic rats.

The OGT curve of the diabetic rats exhibited a significant (*p* < 0.01; LSD) elevation at all tested periods (0, 30, 60, 90, and 120 min) after oral glucose intake compared with that of normal animals. The oral administration of *B*. *aegyptiaca* fruit and seed extracts to diabetic rats induced a potential amelioration of elevated values at all tested points of the OGT curve. However, the seed extract was more potent at 30 and 60 min after oral glucose intake (Figure 3). The F-probability of OGTT data indicated that the effect between groups was very highly significant (*p* < 0.01).

### 3.3. Effects on Serum Insulin and C-Peptide Levels

As indicated in Table 3, the diabetic rats showed a highly significant decrease (*p* < 0.01; LSD) in the insulin and C-peptide levels in serum. The treatments of the diabetic rats with the fruit and seed extracts caused a highly significant increase (*p* < 0.01; LSD) in these lowered levels. The diabetic rats treated with the *B*. *aegyptiaca* fruit extract exhibited no significant effects on the insulin and C-peptide levels in comparison with the diabetic rats treated with the *B*. *aegyptiaca* seed extract. However, the effects of the fruit extract was more potent in increasing the serum insulin levels. The F-probability indicated that the general effect between groups was very highly significant (*p* < 0.01).

### 3.4. Effect on HOMA-IR Cell Function, HOMA-IS, and HOMA-β Cell Function

In diabetic rats, the HOMA-IS and HOMA-β cell functions were highly significantly (*p* < 0.01; LSD) decreased, whereas HOMA-IR was highly significantly (*p* < 0.01; LSD) increased. The treatment of diabetic rats with *B*. *aegyptiaca* fruit and seed extracts induced a highly significant increase in HOMA-β cell function and HOMA-IS. In contrast, HOMA-IR was highly significantly decreased after the treatments with the *B*. *aegyptiaca* fruit and seed extracts. 

Although the effects of the *B*. *aegyptiaca* fruit and seed extracts on HOMA-IR and HOMA-IS were more or less similar, the effects of the fruit extract on HOMA-β cell function was more potent than that of the seed extract (Table 4). One-way ANOVA revealed that the effect between groups on the HOMA-IR, HOMA-IS, and HOMA-β cell function was very highly significant (*p* < 0.01; F-probability).

### 3.5. Effects on Liver Glycogen Content and Glucose-6-phospatase and Glycogen Phosphorylase Activities

The diabetic rats exhibited a highly significant (*p* < 0.01; LSD) depletion in liver glycogen content and a highly significant (*p* < 0.01; LSD) elevation in liver glucose-6-phosphatase and glycogen phosphorylase activities. The treatments with the *B*. *aegyptiaca* fruit and seed extracts highly significantly improved (*p* < 0.01; LSD) the reduced liver glycogen content of the diabetic rats and the elevated glucose-6-phosphatase and glycogen phosphorylase activities (Table 5). One-way ANOVA revealed that the effect between groups on liver glycogen content and glucose-6-phosphatase and glycogen phosphorylase activities was very highly significant (*p* < 0.01; F-probability).

### 3.6. Effects on Serum Lipid Profile

The total cholesterol, triglycerides, LDL-cholesterol, vLDL- cholesterol, and FFA levels in serum exhibited a highly significant elevation (*p* < 0.01; LSD) in diabetic rats compared with those in the normal group. The treatment of diabetic rats with the *B*. *aegyptiaca* fruit and seed extracts produced a highly significant improvement in the altered lipid profile in the serum. Moreover, the HDL- cholesterol level was affected in an inverse pattern, as it was highly significantly decreased (*p* < 0.01; LSD) in diabetic rats. 

Conversely, the treatment with the fruit extract induced a significant increase (*p* < 0.01; LSD) compared with the diabetic control, whereas the treatment with the seed extract had no significant effect (*p* > 0.05; LSD) (Table 6). The seed extract was more effective in decreasing the elevated total cholesterol and triglyceride levels in diabetic rats than the fruit extract, whereas the fruit extract was more potent in decreasing the elevated LDL-cholesterol, vLDL-cholesterol, and FFA levels and increasing the lowered HDL-cholesterol level (Table 6). The F-probability revealed that the effect on serum lipid profile between groups was very highly significant (*p* < 0.01).

### 3.7. Effect on Insulin Receptor β-Subunit

**Figure 4:**
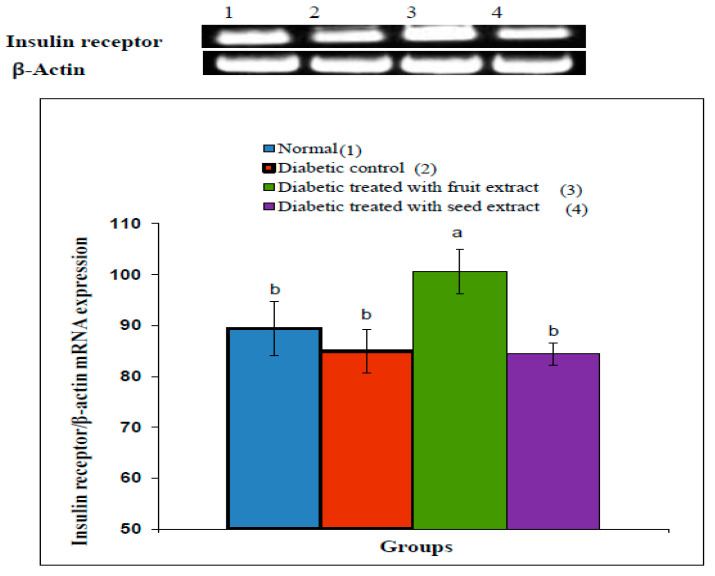
Effects of the *B*. *aegyptiaca* fruit and seed extracts on the adipose mRNA expression of insulin receptor β-subunit relative to β-actin in NA/STZ-administered rats. The means, which have different superscript symbols (a and b), were significantly different at *p <* 0.05. The number of detected samples in each group was three.

The densitometric analysis of the electrophoretogram showed a highly significant (*p* < 0.01; LSD) decrease in the mRNA expression of the adipose tissue insulin receptor β-subunit in diabetic rats compared with that in the normal group. The treatment of diabetic rats with the *B. aegyptiaca* fruit extract produced a highly significant (*p* < 0.01; LSD) amelioration of the insulin receptor β-subunit mRNA expression (Figure 4), whereas the treatment with the *B. aegyptiaca* seed extract did not show a significant effect (*p* > 0.05; LSD). One-ANOVA indicated that the effect between groups on the mRNA expression of insulin receptor β-subunit was highly significant (*p* < 0.01; F-probability).

### 3.8. Effect on Oxidative Stress and Anti-Oxidant Defense Parameters

The liver LPO exhibited a highly significant (*p* < 0.01; LSD) increase in diabetic rats compared with that in normal rats. The treatment with the *B*. *aegyptiaca* fruit and seed extracts in diabetic rats resulted in a highly significant (*p* < 0.01; LSD) amelioration in LPO; the effects of the fruit extract were the most potent.

The GSH content as well as the GPx, GST, and SOD activities showed a highly significant (*p* < 0.01; LSD) decline in diabetic control rats compared with those in the normal rats. The treatments with the *B*. *aegyptiaca* fruit and seed extracts successfully improved the GSH content and GPx, GST (*p* < 0.01; LSD), and SOD activities (*p* < 0.05; LSD) (Table 7). While the effects of the fruit and seed extracts on GSH content and anti-oxidant enzyme activities were more or less similar, the seed extract was more potent in decreasing the elevated LPO.

The F-probability indicated that the effect on the liver LPO, GSH content, and GPx, GST, and SOD activities between groups was very highly significant (*p* < 0.01; F-probability)

### 3.9. Histological Changes in the Pancreas

**Figure 5:**
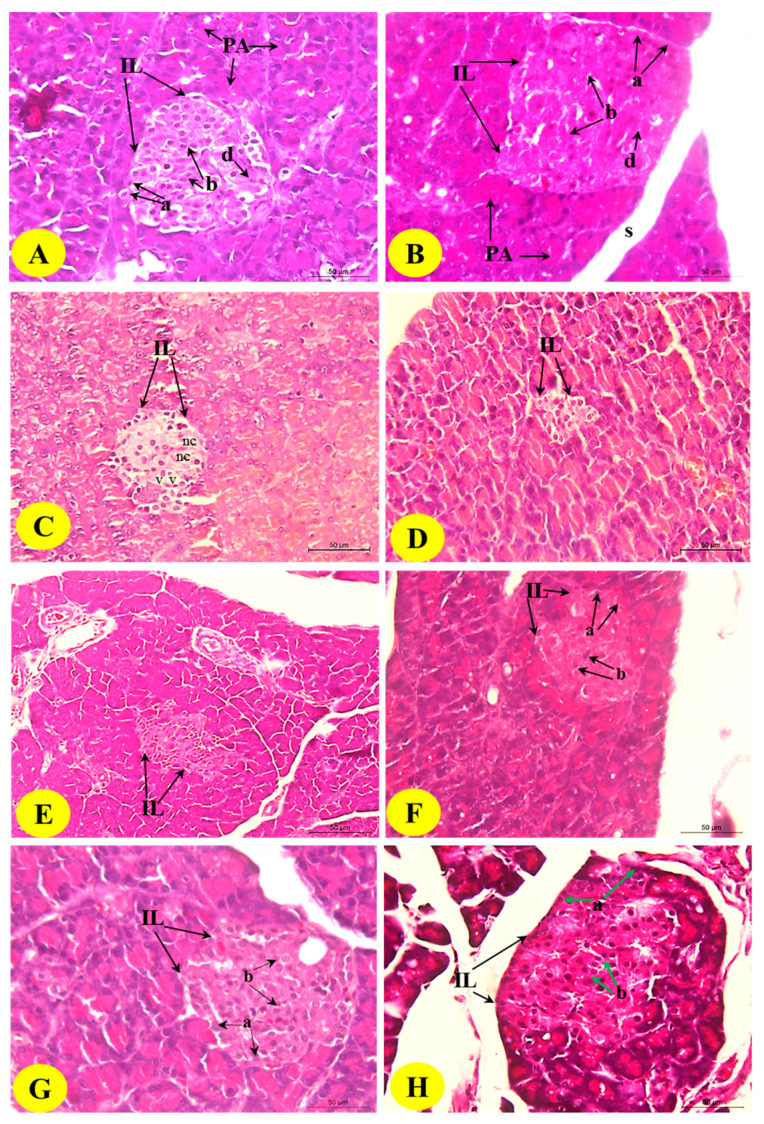
Photomicrographs of the H&E-stained pancreas of the normal (**A**,**B**), diabetic control (**C**,**D**), and diabetic rats treated with the *B*. *aegyptiaca* fruit (**E**,**F**) and seed extracts (**G**,**H**). Photomicrographs A and B show normal pancreatic lobules consisting of pancreatic acini and intact islets of Langerhans (IL) with alpha (a), beta (b), and delta cells. Photomicrographs C and D show the islets of Langerhans with the reduced size and smaller number of the cells; the islets exhibited necrosis (nc) and vacuolations (v) (H&E stain: Scale bar = 50 µm). Photomicrographs E–H show considerable improvements in the islets of Langerhans with a greater increase in the islet size and the number of islet cells.

The histopathological examination of the control pancreas sections showed closely packed lobules of the pancreatic acini. The islet of Langerhans is composed of numerous compactly arranged cells (alpha, beta, and delta cells) occurring as dense cords (Figure 5; Photomicrographs A and B). The diabetic control revealed the histopathological changes of endocrine portions represented by a marked decrease in the size of the islets of pancreas (pancreatic shrinkage) and decreased number of the cells. 

The islets also exhibited cytoplasmic vacuolations (v) and necrosis (nc) (Figure 5; Photomicrographs C and D). The islets of Langerhans exhibited nearly normal and organized architecture, and the necrotic and degenerative changes were markedly improved in rats treated with the *B*. *aegyptiaca* fruit (Figure 5; Photomicrographs E and F) and seed extracts (Figure 5; Photomicrographs G and H) compared with those in the diabetic control rats; the seed extract was found to be the most potent.

## 4. Discussion

DM is a group of metabolic diseases characterized by chronic hyperglycemia due to defects in insulin secretion, insulin action, or both [47]. STZ is known for its selective pancreatic islet β-cell cytotoxicity and has been frequently used to induce DM in animals. NA/STZ-induced DM is selected to be a model of type 2 DM (T2DM) to assess the effects of the *B*. *aegyptiaca* fruit and seed aqueous extracts. In this model, when NA is injected prior to the administration of STZ, the severity of DM will be reduced to a certain extent, leading to a T2DM-like condition with deteriorated IS [20,35,48,49,50].

Several traditional medicines have been discovered for DM. Isolated substances and extracts isolated from various natural resources particularly plants have always been a rich arsenal for the control and treatment of DM problems and complications [9]. Plants have rich sources of antidiabetic as well as anti-hyperlipidemic and anti-oxidant substances, such as flavonoids, amino acids, gallotannins, and many other related polyphenols [51]. *B*. *aegyptiaca* is a plant widely used as a hypoglycemic agent in Egyptian folkloric medicine [15]. However, only few studies have investigated the antidiabetic effects and the mechanisms of action of *B*. *aegyptiaca,* especially its seeds.

In the present study, GC-MS data presented in Table 1 and Table 2 revealed the presence of several active ingredients. In the *B*. *aegyptiaca* fruit aqueous extract, 3,4,6-tri-O-methyl-d-glucose (52.55%) and triethylphosphine (9.31%) were the most abundant, whereas in the seed aqueous extract, 9,12-octadecadienoic acid (Z,Z)- (38.27%), 8-dodecen-1-ol, (Z)- (15.09%), 2,3-dihydroxypropyl ester (11.47%), and H-cyclopenta [b]quinoxaline-1,2,3trione (11.39%) were the major components. 

Many of the constituting ingredients of the *B*. *aegyptiaca* fruit and seed aqueous extracts exhibit many biological activities. Of these, 9,12-octadecadienoic acid (Z,Z)- have anti-inflammatory, hepatoprotective, cancer preventive, and hypocholesterolemic effects [20,52,53]. The derivative 9-octadecenoic acid was reported to have antitumor and anti-inflammatory activities [53]. Isothiocyanates have been demonstrated to have both anti-inflammatory and antioxidant activities [54].

OGTT is a well-known and common assay used to determine the anti-hyperglycemic activity of antidiabetic agents [55]. OGTT is considered the gold standard test for the diagnosis of DM by the World Health Organization [56]. The data in the present study revealed a marked increase in the serum glucose levels of the diabetic groups compared with those of the normal rats. These findings are consistent with those of Akhani et al. [57], Ahmed [58], Schaalan et al. [59], Ahmed et al. [60], Ahmed et al. [61], and Ali et al. [62]. 

The increase in glucose level may be due to the decreased glucose consumption in the peripheral, muscle, and adipose tissues [63] and increased glycogen breakdown [64], gluconeogenesis, and production of hepatic glucose [65]. Furthermore, Powers confirmed that IR in T2DM induces an increase in blood glucose because of the same causes [66]. In the present study, the treatment of diabetic rats with the *B*. *aegyptiaca* fruit or seed extracts caused a potential improvement in OGT. The decrease in the elevated serum glucose levels is in accordance with the results of Zaahkouk et al. [67], Helal et al. [68], and Al-Malki et al. [69] who verified the anti-hyperglycemic effects of the *B*. *aegyptiaca* fruit. 

The ameliorative effects of the *B*. *aegyptiaca* extracts may be associated with insulinomimetic activities [70], stimulation and potentiation of insulin secretion, increased affinity of insulin receptors [71], improved concentration of hepatic glycogen, accelerated glucose metabolism, reduced production of intestinal glucosidase, and decreased gluconeogenesis of the liver [15]. These actions may be attributed to the active constituting ingredients found in the *B*. *aegyptiaca* fruit and seed extracts. In this regard, a previous publication found that *B*. *aegyptiaca* may contain interketones, organic constituents, rutin, and oils (fatty acids and volatile oils) present in the internal kernel according to the phytochemical investigation [72]. 

Furthermore, Baragob et al. attributed the hypoglycemic effects of the aqueous extract to its constituents, such as saponins, rutin, and organic constituents [73]. In the present study, the GC-MS analysis indicated the presence of many organic ingredients, which have several biological activities, including antidiabetic potencies.

In the present study, the NA/STZ-induced diabetic rats exhibited a significant decrease in serum insulin and C-peptide levels. It is important to note the relationship between decreased insulin and C-peptide levels in diabetic rats and decreased size and number of the islets of Langerhans that have a decreased number of β-cells, necrosis, and vacuolations. These decreases are also correlated with the calculated HOMA**-**β cell function. 

The administration of the *B*. *aegyptiaca* fruit and seed aqueous extracts produced a significant increase in serum insulin and C-peptide levels of diabetic rats, and this finding is consistent with that of Abou Khalil et al. [74], El-Bayomy et al. [75], and Hassan [76]. In this regard, Abdel-Moneim [70] hypothesized that the hypoglycemic action of the *B*. *aegyptiaca* aqueous extract stimulated the β-cells of the pancreatic islets to secrete insulin, potentiate glucose-stimulated insulin secretion, and increase the number and sensitivity of insulin receptors and post-receptor effects in peripheral tissues. 

Furthermore, the *B*. *aegyptiaca* seeds contain diosgenin [77], which may be useful in ameliorating the glucose metabolic disorder related with DM and obesity as reported by Ulbricht et al. [78]. C-peptide is formed during insulin biosynthesis, and the two peptides, insulin and C-peptide, are then released to the circulation in equal amounts [79]. An increase in C-peptide levels in diabetic rats treated with *B*. *aegyptiaca* corresponds well with the increase in insulin secretion (endogenous secretion), which is possibly due to the regeneration of the β-cells of the islets of Langerhans. 

This association is demonstrated in the present study by the significant increase in HOMA-β cell function and marked improvement in the histological architecture and number of β cells of the pancreatic islets as a result of the treatment with the *B*. *aegyptiaca* fruit and seed extracts.

The level of liver glycogen may be regarded as the best marker for evaluating the anti-hyperglycemic activity of any drug [80]. The present study showed that the diabetic rats exhibited a significant depletion of liver glycogen content correlated with a marked increase of glucose-6-phosphatase and glycogen phosphorylase activities. These results are consistent with those of Sundaram et al. [81] and Mahmoud et al. [82]. In the present study, the treatment with the *B*. *aegyptiaca* fruit and seed extracts significantly improved the lowered liver glycogen content and the elevated hepatic glucose-6-phosphatase and glycogen phosphorylase activities. 

**Figure 6:**
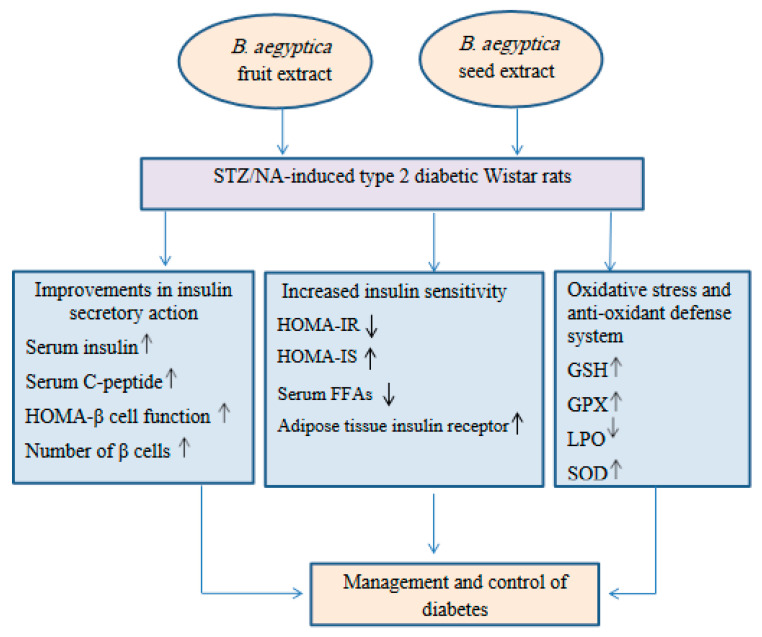
Schematic illustrative figure depicting the suggested hypothesis of the modes of action of the *B*. *aegyptiaca* fruit and seed aqueous extracts.

These ameliorations may be secondary to the increase in the insulin levels in the blood and the enhanced IS. These results are consistent with those of Helal et al. [68] who found that the regeneration of β-cells led to an increase in the insulin level and improvement in IS, both of which can lower the glucose levels in the blood. The present results support this finding as the treatment of diabetic rats with the *B*. *aegyptiaca* fruit and seed extracts significantly increased the HOMA-β cell function and HOMA-IS but significantly decreased HOMA-IR (Figure 6).

Hypoinsulinemia and IR clearly shown in untreated diabetic rats are considered the main cause of the recorded dyslipidemia represented by hypertriglyceridemia and hypercholesterolemia associated with the increased production of vLDL cholesterol and LDL cholesterol and decreased HDL cholesterol level. These findings are consistent with those of Abdel-Moneim et al. [83] who reported a marked increase in the levels of serum triglycerides, cholesterol, and LDL cholesterol of diabetic rats. This increase may be due to a decrease in lipoprotein lipase function caused by insulin deficiency [84]. 

In addition, Goodman and Gilman [85] reached the same results, which were explained by inhibition of lipoprotein lipase transcription inside the capillary endothelium as a result of insulin deficiency. The data of the present study are consistent with those of Harvey and Ferrier [86] who reported that the metabolic abnormalities of T2DM as a result of IR lead to dyslipidemia in the liver where fatty acids are converted into triacylglycerol, which, in turn, are packaged and secreted in vLDL. Both accumulations of lipids, especially triglycerides, and reduced anti-oxidant activity contribute to the development of oxidative stress in diabetic rats [87].

The results of the present study are consistent with those of previous studies [88,89,90], which revealed a reduction in the HDL cholesterol level in the diabetic control group. The present results of the serum lipid profile are consistent with the findings of Stanfield [91] who stated that DM increases the number of LDL particles that transport lipids, including cholesterol to peripheral tissues, and decreases the number of HDL particles, which transport lipids and cholesterol to the liver. 

Simultaneously, the elevated serum triglyceride level in the diabetic group of the present study may be related to the decreased clearance and increased production of endogenously synthesized main triglyceride transporters [92]. Moreover, the expansion of the cholesterol pool in DM could be explained by the increased input into the system by accelerating the synthesis of intestinal cholesterol or by increasing the rate of absorption of intestinal cholesterol [93].

Indeed, the improvement in dyslipidemia through *B*. *aegyptiaca* treatment may be related to the increased level and sensitivity of insulin. The present results are not in concordance with those of Matter and Helal [94] who reported that the level of serum triglycerides and cholesterol was not significantly different when compared with the control group after treatment with the *B. aegyptiaca* seed extract. 

In accordance with the findings of Abd El-Rahman and Al-ahmari [95], the improvement of lipid profile may be due to the presence of saponins in its extract, indicating antihypercholesterolemic and hypoglycemic activities. Moreover, diosgenin in the *B*. *aegyptiaca* seed kernels plays an important role in the regulation of cholesterol metabolism [96]. In the present study, the GC-MS analysis indicated the presence of many organic ingredients, which have several biological activities, including antihypercholesterolemic properties.

The elevated serum FFA level recorded in diabetic rats in the present study is consistent with that estimated in many preceding studies [35,62,97,98]. The elevated release of FFAs from the adipose tissue can be attributed to the lipolysis of visceral adipose depots; this effect can result in IR, excessive endogenous glucose formation, and progression to T2DM [99]. Thus, decreasing the plasma FFA level is recommended as a method for IR prevention and treatment [100]. 

During the treatment of diabetic animals with *B*. *aegyptiaca* aqueous extracts, a reduction in the amount of serum FFA levels that could be associated with the insulin-sensitizing activity of the extract was observed [101]. Furthermore, several studies have found significantly low levels of resistin mRNA in the adipose tissue in various obese mouse models, such as db/db or high-fat diet–induced obesity, as well as in rat models with IR [102].

The present study showed a significant reduction in the mRNA expression of insulin receptor β-subunit in the adipose tissues of the diabetic group in comparison with that of the normal group. This result is consistent with that of Ali et al. [62] and Abdel Aziz et al. [51] who demonstrated that the mRNA expression of insulin receptor β-subunit was significantly decreased in NA/STZ-induced diabetic rats. This effect provides evidence of the presence of IR and impaired IS in such animal models, which in turn is a suitable model of T2DM. In the present study, the treatment with the *B*. *aegyptiaca* fruit aqueous extract produced a significant increase in the mRNA expression of insulin receptor β-subunit reflecting the ability of this extract to reduce IR and enhance IS in the adipose tissues (Figure 6).

Oxidative stress is an important factor in DM etiology and pathogenesis, causing interactions with polyunsaturated fatty acids that contribute to LPO [103]. According to Randle’s theory on glucose-fatty acids [104], the excessive release of free fatty acids from the adipose tissue for oxidation induces the production of metabolites that prevent tissue use of glucose. Such metabolites are reactive oxygen species and hydrogen peroxide, which are involved with the glucose-fatty acid process [105]. 

Belfort et al. [106] showed that the increase in plasma FFA caused a dose-dependent inhibition of insulin-stimulated glucose disposal and insulin signaling in the skeletal muscle of lean healthy individuals. The present findings showed a significant increase in LPO in the liver. Additionally, the GSH level and anti-oxidant enzyme defenses decreased simultaneously in the liver of diabetic rats. These findings are consistent with those of several studies [35,89,89,107,108]. GSH plays a multifaceted role in the defense against anti-oxidants. It actively scavenges free radicals or indirectly detoxifies reactive species via GST and GPx [109]. 

The treatment of diabetic rats with *B*. *aegyptiaca* extracts significantly decreased MDA, which is attributed to the increased levels of anti-oxidants that fight free radicals [110] and markedly increased GSH level and SOD and GPx activities. Thus, it is worth noting that the improvement in the glycemic state, lipid profile, and insulinotropic and insulin-sensitizing effects is associated with the suppression of oxidative stress and enhancement of the anti-oxidant defense system. 

This indicated that the decrease in oxidative stress and enhancement of the anti-oxidant defense system may have an important role in the improvement of the architecture and tissue IS of the pancreatic islets, which in turn result in the effective management of diabetes. These findings are consistent with those of Hassanin et al. [111] who indicated that *B*. *aegyptiaca* exerted hypoglycemic, hypolipidemic, and insulinotropic actions associated with the reduction in oxidative stress, enhancement in the anti-oxidant defense system, and reduced apoptosis in pancreatic β-cells.

In conclusion, the *B*. *aegyptiaca* fruit and seed aqueous extracts have potent antidiabetic potencies, which may be mediated via improvements in the insulin secretory response, β-cell function, tissue IS, and anti-oxidant defense system (Figure 6).

## Figures and Tables

**Table 1:** Chemical groups and compounds present in the *B*. *aegyptiaca* fruit aqueous extract.

Number	(RT) Retention Time	Compound Name	Area% (Higher than 1%)
1	2.123	Hydrazine, 1,1-dimethyl-	4.31
2	3.008	Butanal, 2-methyl-	3.04
3	3.043	Butanal, 2-methyl-	1.89
4	3.363	Propanoic acid, propyl ester	3.41
5	3.907	Glyceraldehyde	5.39
6	4.250	2-Furanmethanol	2.55
7	4.645	Isopropyl isothiocyanate	2.64
8	4.889	Triethylphosphine	9.31
9	9.454	2,4(3H,5H)-Furandione	1.46
10	9.744	Pyridine, 4-chloro-2,6-dimethyl-	0.96
11	18.561	3,4,6-Tri-O-methyl-d-glucose	52.55
12	21.667	Perhydrohistrionicotoxin-2-thione, 2-depentyl-	2.07
13	23.758	Propyl 11,12-methylene-octadecanoate	3.06

**Table 2:** Chemical groups and compounds present in the *B*. *aegyptiaca* seed aqueous extract.

Number	Retention Time	Compound Name	Area% (Higher than 1%)
1	4.263	Butane, 2-methyl-	1.67
2	20.538	-3,4,6Tri-O-methyl-d-glucose	2.59
3	24.018	H-Cyclopenta [b]quinoxaline-1,2,3trione	11.39
4	25.505	-9,12Octadecadienoic acid (Z,Z)-	2.47
5	25.568	13-Octadecenoic acid, methyl ester	1.62
6	26.152	9,12-Octadecadienoic acid (Z,Z)-	38.27
7	26.192	8-Dodecen-1-ol, (Z)-	15.09
8	26.281	9,12-Octadecadienoic acid (Z,Z)-, 2,3-dihydroxypropyl ester	11.47
9	26.339	Octadec-9-enoic acid	6.06
10	26.371	Benzoic acid, 4-(4-hydroxybenzylidenamino)-, propyl ester	6.79

**Table 3:** Effects of the *B*. *aegyptiaca* fruit and seed extracts on the serum insulin and C-peptide levels in NA/STZ-induced diabetic rats.

	Parameter	Insulin (μIU/mL)	C-Peptide (pg/mL)
Group			
Normal		2.56 ± 0.51 ^a^	4.7 ± 0.13 ^a^
Diabetic control		1.00 ± 0.78 ^c^	1.19 ± 0.13 ^c^
Diabetic treated with fruit extract		1.71 ± 0.11 ^b^	3.47 ± 0.44 ^b^
Diabetic treated with seed extract		1.56 ± 0.11 ^b^	3.50 ± 0.22 ^b^

- Data were expressed as the mean ± SE. The number of animals in each group was six. - Means, which have different superscript symbols (a, b, and c), were significantly different at *p <* 0.05.

**Table 4:** Effects of the *B*. *aegyptiaca* fruit and seed extracts on HOMA-IR cell function, HOMA-IS, and HOMA-β cell function in NA/STZ-induced diabetic rats.

	Parameter	HOMA-IR	HOMA-IS	HOMA-β Cell Function
Group				
Normal		0.37 ± 0.03 ^c^	55.48 ± 2.23 ^a^	55.50 ± 1.42 ^a^
Diabetic control		0.51 ± 0.04 ^a^	45.50 ± 0.66 ^b^	2.71 ± 0.17 ^d^
Diabetic treated with fruit extract		0.40 ± 0.03 ^b^	55.00 ± 1.23 ^a^	11.20 ± 0.90 ^b^
Diabetic treated with seed extract		0.45 ± 0.03 ^b^	53.33 ± 1.03 ^a^	7.80 ± 0.39 ^c^

- Data were expressed as the mean ± SE. The number of animals in each group was six. - Means, which have different superscript symbols (a, b, c, and d c), were significantly different at *p <* 0.05.

**Table 5:** Effects of the *B*. *aegyptiaca* fruit and seed extracts on liver glycogen content and glucose-6-phosphatase and glycogen phosphorylase activities in NA/STZ-induced diabetic rats.

	Parameter	Liver Glycogen (mg P liberated/g Tissue/h)	Glucose-6-phosphatase (mg Pi liberated/g Tissue/h)	Glycogen Phosphorylase (mg Pi liberated/g Tissue/h)
Group				
Normal		54.55 ± 3.60 ^a^	25.79 ± 1.53 ^b^	22.78 ± 3.09 ^b^
Diabetic control		8.10 ± 1.66 ^b^	67.42 ± 3.38 ^a^	41.85 ± 6.08 ^a^
Diabetic treated with fruit extract		42.75 ± 2.91 ^a^	28.02 ± 0.41 ^b^	25.45 ± 4.95 ^b^
Diabetic treated with seed extract		50.51 ± 3.60 ^a^	31.45 ± 2.28 ^b^	15.22 ± 2.20 ^b^

- Data were expressed as the mean ± SE. The number of animals in each group was six. - Means, which have different superscript symbols (a, b, and c), were significantly different at *p <* 0.05.

**Table 6:** Effects of the *B*. *aegyptiaca* fruit and seed extracts on serum lipid profile and FFA level in NA/STZ-induced diabetic rats.

	Parameter	Total Cholesterol (mg/dL)	Triglycerides (mg/dL)	LDL-Cholesterol (mg/dL)	HDL- Cholesterol (mg/dL)	vLDL- Cholesterol (mg/dL)	FFAs (mg/dL)
Group							
Normal		42.70 ± 1.34 ^c^	35.66 ± 2.25 ^c^	5.10 ± 2.10 ^c^	31.30 ± 2.30 ^a^	7.11 ± 0.33 ^c^	5.10 ± 0.86 ^b^
Diabetic control		79.40 ± 9.48 ^a^	80.25 ± 15.30 ^a^	37.48 ± 6.03 ^a^	24.80 ± 2.80 ^b^	16.08 ± 3.00 ^a^	18.70 ± 2.7 ^a^
Diabetic treated with fruit extract		59.70 ± 9.30 ^b^	53.70 ± 2.83 ^b^	13.18 ± 4.76 ^b^	31.00 ± 2.10 ^a^	8.11 ± 1.05 ^c^	5.10 ± 0.70 ^b^
Diabetic treated with seed extract		51.80 ± 9.70 ^bc^	41.25 ± 5.34 ^c^	15.94 ± 7.90 ^b^	27.10 ± 0.80 ^b^	10.71 ± 0.056 ^b^	6.75 ± 1.80 ^b^

- Data were expressed as the mean ± SE. The number of animals in each group was six. - Means, which have different superscript symbols (a, b, and c), were significantly different at *p <* 0.05.

**Table 7:** Effects of the *B*. *aegyptiaca* fruit and seed extracts on liver LPO, GSH content, and GPx, GST, and SOD activities in NASTZ-induced diabetic rats.

	Parameter	LPO (nmole MDA/100 mg Tissue/h)	GSH (nmole/100 mg Tissue)	GPx (mU/100 mg Tissue)	GST (U/100mg Tissue)	SOD (U/g Tissue)
Group						
Normal		67.17 ± 1.25 ^bc^	13.30 ± 1.63 ^a^	42.60 ± 5.1 ^a^	12.65 ± 3.6 ^b^	16.78 ± 1.59 ^a^
Diabetic control		111.39 ± 7.57 ^a^	6.10 ± 1.60 ^c^	17.20 ± 5.1 ^c^	6.57 ± 1.65 ^c^	10.31 ± 1.58 ^c^
Diabetic treated with fruit extract		68.75 ± 3.80 ^b^	12.10 ± 1.54 ^ab^	30.80 ± 4.6 ^b^	16.54 ± 0.86 ^a^	13.46 ± 2.55 ^b^
Diabetic treated with seed extract		52.95 ± 5.821 ^c^	11.20 ± 1.5 ^b^	31.00 ± 2.5 ^b^	16.43 ± 0.85 ^a^	13.26 ± 2.08 ^b^

- Data were expressed as the mean ± SE. The number of animals in each group was six. - Means, which have different superscript symbols (a, b, and c), were significantly different at *p <* 0.05.

## Data Availability

The data presented in this study are available in this manuscript.

## Abbreviations

*B*. *aegyptiaca*: *Balanites aegyptiaca*; b.w.: body weight; DM: Diabetes mellitus; ELISA: sandwich enzyme-linked immunosorbent assay; FFA: free fatty acid; GC-MS: Gas chromatography–mass spectrometry; GPx: glutathione peroxidase; GST: glutathione-S-transferase; H&E: haematoxylin and eosin; HDL: high density lipoprotein; HOMA-IR: homeostatic model assessment-insulin resistance; HOMA-IS: homeostatic model assessment- insulin sensitivity; HOMA-β cell function: homeostatic model assessment-β cell function; IDF: International Diabetes Federation; GSH: reduced glutathione; IP: intraperitoneal; IR: insulin resistance; IS: insulin sensitivity; LDL: low density lipoprotein; LPO: lipid peroxidation; LSD: least significant difference; MDA: malondialdehyde; mRNA: messenger RNA; OGT: oral glucose tolerance; OGT: oral glucose tolerance; OGTT: oral glucose tolerance test; RNA: Ribonucleic acid; RT-PCR: reverse transcription-polymerase chain reaction; SOD: superoxide dismutase; STZ: streptozotocin; T2DM: Type 2 DM; and vLDL: very low density lipoprotein.
